# The impact of metabolic syndrome severity on racial and ethnic disparities in Metabolic Dysfunction-Associated Steatotic Liver Disease

**DOI:** 10.1371/journal.pone.0299836

**Published:** 2024-03-15

**Authors:** Mohamed I. Elsaid, John F. P. Bridges, Khalid Mumtaz, Na Li, Lindsay Sobotka, Vinod K. Rustgi, Electra D. Paskett

**Affiliations:** 1 Department of Biomedical Informatics, College of Medicine, The Ohio State University, Columbus, Ohio, United States of America; 2 Center for Biostatistics, College of Medicine, The Ohio State University, Columbus, Ohio, United States of America; 3 Department of Internal Medicine, Division of Medical Oncology, College of Medicine, The Ohio State University, Columbus, Ohio, United States of America; 4 Department of Health, Behavior and Society, The Johns Hopkins Bloomberg School of Public Health, Baltimore, Maryland, United States of America; 5 Division of Gastroenterology, Hepatology, & Nutrition, College of Medicine, The Ohio State University, Columbus, Ohio, United States of America; 6 Division of Gastroenterology and Hepatology, Rutgers Robert Wood Johnson Medical School, New Brunswick, New Jersey, United States of America; 7 Center for Liver Diseases and Masses, Rutgers Robert Wood Johnson Medical School, New Brunswick, New Jersey, United States of America; 8 Division of Population Sciences, The Ohio State University Comprehensive Cancer Center, Columbus, Ohio, United States of America; 9 Department of Internal Medicine, Division of Cancer Prevention and Control, College of Medicine, The Ohio State University, Columbus, Ohio, United States of America; Dartmouth Health, UNITED STATES

## Abstract

**Background & aims:**

Previous studies have examined the effects of metabolic syndrome (MetS) rather than its severity on race and ethnic disparities in Metabolic Dysfunction-Associated Steatotic Liver Disease (MASLD). We used the MetS severity score, a validated sex-race-ethnicity-specific severity measure, to examine the effects of race/ethnicity on the association between MetS severity and MASLD.

**Methods:**

This study included 10,605 adult participants from the Third National Health and Nutrition Examination Survey. The MASLD diagnosis was based on ultrasound findings in patients without excessive alcohol intake or other liver diseases. MetS severity Z-scores were calculated and stratified into four categories low (1^st^-50^th^), moderate (>50^th^-75^th^), high (>75^th^-90^th^), and very high (>90^th^+)]. Multivariable adjusted logistic regression models with complex survey methods were used to test the effect of MetS severity on MASLD.

**Results:**

The age-adjusted MASLD prevalence was 17.4%, 25.7%, 42.5, and 54.9% in adults with mild, moderate, high, and very high MetS severities, respectively (P-trend <0.001). MetS severity was significantly higher in patients with MASLD than in those without [mean percentile 60^th^ vs. 44^th^, P<0.001]. Among patients with MASLD, Mexican-American and Black non-Hispanic females had significantly higher age-adjusted MetS severity (68^th^ and 61^st^, respectively) than White non-Hispanic females 54^th^, while Black non-Hispanic males had significantly lower MetS severity (56^th^) than White non-Hispanic males (70^th^) (P-Interaction = 0.02). Adults with high and very high MetS severity had 2.27 (95% CI:1.70 to 3.03) and 3.12 (95% CI:2.20 to 4.42), respectively, higher adjusted odds of MASLD than those with mild MetS severity.

**Conclusions:**

Racial/ethnic disparities in MetS severity play a pivotal role in the risk of MASLD. Our findings highlight the potential clinical utility of the MetS severity score in identifying at-risk individuals, which will help guide targeted prevention and tailoring management strategies to mitigate the MASLD burden.

## 1. Introduction

The growing prevalence of sedentary behavior, unhealthy diets, and obesity-induced metabolic syndrome has resulted in an unprecedented surge in the global burden of Metabolic Dysfunction-Associated Steatotic Liver Disease (**MASLD**) [1], formerly known as nonalcoholic fatty liver disease (NAFLD) [2, 3]. MASLD is recognized as the most common cause of chronic liver disease (CLD), affecting an estimated 24% of adults globally [4, 5]. MASLD is characterized by detectable fatty deposits in the liver without chronic viral hepatitis, other etiologies of CLD, steatosis-inducing medications, or excessive alcohol consumption. Metabolic dysfunction-associated steatohepatitis (MASH), formerly known as nonalcoholic steatohepatitis (MASH), is a progressive form of MASLD, as it describes chronic inflammation of hepatocytes with or without fibrosis [6–8]. While most MASLD patients do not require clinical interventions, MASH increases the risk of progression to advanced CLD, including cirrhosis, decompensated cirrhosis, and hepatocellular carcinoma [9, 10].

Due to obesity-induced insulin resistance’s primary function in promoting hepatic steatosis, MASLD is the most common hepatic manifestation of Metabolic Syndrome (MetS) [4, 7, 11–14]. MetS comprises a group of metabolic abnormalities associated with an increased risk of insulin resistance and cardiovascular disease (CVD). The five clinical features of MetS are atherogenic *hyperglycemia*, *dyslipidemia*, *hypertriglyceridemia*, hypertension, and central obesity [15]. The National Heart, Lung, and Blood Institute guidelines for diagnosing MetS utilize a "harmonization definition" of this syndrome. As such, MetS is diagnosed based on a patient presenting abnormalities that exceed prespecified cutoff values for three of the five clinical features of MetS–metabolic abnormalities [16].

Several epidemiological studies have shown that global prevalence varies considerably, with the Middle East at 32%, South America at 30%, and Asia, Europe, and Africa at 27%, 24%, and 13%, respectively [17]. According to a meta-analysis of 34 studies with more than 360,000 unique patients, strong disparities in MASLD prevalence, severity, and prognosis exist between race and ethnic groups within the United States (US) [18]. As such, several studies suggest that Hispanics have the highest incidence of MASLD, with obesity emerging as a central factor in this population. In these studies, African Americans had the lowest MASLD incidence [19–24]. Genetic and metabolic factors have been suggested to underlie the observed racial disparities [25–28], as have incidence rates of insulin resistance and serum triglyceride concentrations [29], but conclusive evidence in support of any model explaining those disparities has yet to be uncovered.

Existing evidence on the natural history of MASLD has focused on identifying disease biomarkers, risk factors, and clinical outcomes [11, 30] and has included MetS diagnosis as a dichotomous risk factor (*i*.*e*., present or absent). As the natural history of MASLD is closely related to the component features of MetS, examining the effects of MetS using the current dichotomous definition creates several shortcomings when assessing the effects of MetS on MASLD risk and associated outcomes [31]. First, current guidelines for diagnosing MetS entail the occurrence of any three of the five metabolic abnormalities (*i*.*e*., ten possible combinations of metabolic abnormalities). The dichotomous nature of this classification equally treats the effects of any of the five combinations of metabolic abnormalities on the MASLD risk and associated outcomes. Second, MetS diagnosis involves meeting at least three predefined cutoff points for any of the five metabolic abnormalities and neglects the sole and combined values of all MetS features. Third, dichotomous MetS categorization makes it challenging to study and monitor the clinical implications of MetS progression. Fourth, the binary system for MetS definition does not account for racial and sex disparities in MetS severity and their corresponding effects on the risk of MASLD [32].

Assessing MetS by using a continuous measure of disease severity will provide a comprehensive evaluation of its role in MASLD development and progression. The MetS severity score is a validated clinically accessible sex- and race-ethnicity-specific Z-score that encapsulates the combined effects of the nature and severity of all five metabolic abnormalities among adults in the US [33]. The MetS severity score better represents the traditional MetS classification while adjusting for sex and racial/ethnic disparities in the relationship between MetS and cardiometabolic outcomes. The MetS severity score is significantly correlated with pathophysiological biomarkers of MetS, including the Homeostasis Model for Insulin Resistance (HOMA-IR), C-reactive protein (CRP), uric acid, and adiponectin [33, 34]. Multiple studies have also shown that the MetS severity score is significantly associated with long-term risks of cardiovascular disease, type 2 diabetes mellitus, and coronary heart disease [34–37]. Furthermore, the MetS severity score predicts all-cause and cause-specific mortalities in MASLD [31].

We used the MetS severity score to examine the effects of race/ethnicity on the association between MetS severity and MASLD. Understanding the effects of MetS severity on MASLD occurrence will aid clinicians and healthcare professionals in identifying high-risk individuals and in monitoring disease progression. In addition, the results of this study will assist in developing public health interventions to identify the highest priority groups and optimal prevention strategies based on disease severity.

## 2. Materials and methods

### 2.1. Data source

This cross-sectional study used data from the Third National Health and Nutrition Examination Survey (NHANES III) between 1988 and 1994. The survey was conducted to assess the health status of the US population, focusing on non-Hispanic Blacks, Mexican Americans, and individuals aged 60 years or older. NHANES III utilized a stratified multistage clustered probability design to sample non-institutionalized members of the US population aged two months or older. The survey included cross-sectional examinations, interview questionnaires, and laboratory sample collection. Of all the participants interviewed, 78% completed the physical examination phase of the survey [38]. The Centers for Disease Control and Prevention (CDC) oversaw the recruitment of all study participants and collection of all consent forms. The consent forms were signed by participants in the survey, and they consented to storing blood specimens for future research. The CDC’s ethics review board approved the NHANES III survey protocol. The study was exempted by the Ohio State University Institutional Review Board because the NHANES III database is a publicly available database that meets the criteria for a limited-use dataset.

### 2.2. Study sample

The study sample included all the adult participants with valid hepatic steatosis evaluations. Hepatic steatosis was evaluated by three trained ultrasound readers using video-recorded gallbladder ultrasounds during physical examinations of all NHANES III participants aged 20–74 years. Following the initial assessments, all ultrasound readings were re-evaluated and validated by a certified radiologist specializing in hepatic imaging. Hepatic steatosis images were classified as normal, mild, moderate, or severe. The criteria for grading hepatic steatosis included gallbladder wall definition, liver parenchyma degree of brightness, occurrence of deep beam attenuation, presence of liver-to-kidney contrast, and echogenic walls in the small intrahepatic vessels [39].

Of the 16,573 participants aged 20 years or older who attended the survey examination phase, 14,707 qualified for gallbladder ultrasound reading, of which 13,856 had readable ultrasound images [40]. Participants were excluded from the study if they had missing values for exposure, outcome, alcohol intake, ultrasound images, or any of the covariates included in the adjusted analyses. In addition, participants who identified as "Other" on the race/ethnicity questions were also excluded, as the exposure assessment was only available for non-Hispanic whites, non-Hispanic blacks, and Mexican Americans. After implementing all inclusion and exclusion criteria, the final study sample included 10,605 adult participants.

### 2.3. Exposure

The MetS severity score was the primary exposure variable. The MetS severity score is a validated sex- and race/ethnicity-specific Z-score that captures the relative MetS severity of all five metabolic abnormalities [33]. The score was quantified using Confirmatory Factor Analysis (CFA) with data from the 1999–2010 NHANES databases [33]. All five metabolic features were used in the CFA to construct a summary score that is a continuous representation of the conventional metabolic syndrome characterization. We used individual-level data on high-density lipoprotein (HDL), systolic blood pressure (SBP), waist circumference, triglycerides, and fasting blood glucose to calculate sex- and race/ethnicity-specific MetS severity Z-scores according to the score’s standardized equations [33]. Furthermore, we transformed the MetS severity Z-scores into four percentile-based categories [mild (1^st^-50^th^), moderate (>50^th^-75^th^), high (>75^th^-90^th^), and very high (>90^th^)].

The National Cholesterol Education Program (NCEP) Adult Treatment Panel III (ATP III) guidelines were used to define the traditional MetS diagnosis. As such, MetS diagnosis was defined by the presence of three of five metabolic factors: (1) *hyperglycemia* (*i*.*e*., fasting blood glucose > 100 mg/dl, or pharmacological treatment), (2) *dyslipidemia* (*i*.*e*., fasting HDL cholesterol level less than 40 mg/dl, men, or 50 mg/dl, women, or pharmacological treatment), (3) *hypertriglyceridemia* (*i*.*e*., fasting triglyceride level over 150 mg/dl, or pharmacological treatment), (4) central obesity (*i*.*e*., waist circumference over 40 inches for men, or 35 inches for women), or (5) hypertension (*i*.*e*., systolic blood pressure (SBP) over 130 mmHg, or pharmacological treatment) [15].

### 2.4. Outcome

The primary outcome of the study was the MASLD status. MASLD was identified by the presence of mild, moderate, or severe hepatic steatosis in the absence of excessive drinking (*i*.*e*., more than three alcoholic beverages per day for males or more than two alcoholic beverages per day for females), binge drinking (*i*.*e*., frequent consumption of five or more alcoholic beverages per day), alcohol consumption restrictions due to illness, positive Hepatitis B virus (HBV) surface antigen test, positive Hepatitis C virus (HCV) RNA Test, or iron overload (*i*.*e*., transferrin saturation ≥ 50%). Because metabolic syndrome severity was the main exposure in our study, we did not include cardiometabolic assessment as part of our MASLD definition to avoid potential confounding. Accordingly, our current MASLD definition falls under the cryptogenic steatotic liver disease or possible MASLD categories of the current MASLD nomenclature [1].

### 2.5. Covariates

Confounder selection was based on prior literature and theoretical rationale. The confounders used in the adjusted multivariable analyses included age, sex, race/ethnicity (white non-Hispanics, black non-Hispanics, or Mexican Americans), education level (< high school, high school, or GED; some college or college degree or higher), access to health insurance (yes or no), alcohol intake (never, former, or current), smoking status (never, former, or current), body mass index (Kg/m^2^) [underweight (<18.5), healthy weight (≥18.5–25.0<), overweight (≥25.0–30.0<), or obese (≥30)]; abdominal obesity; physical activity (metabolic equivalents per month); healthy eating index percentile; HOMA-IR; aspartate aminotransferase (AST) to alanine aminotransferase (ALT) ratio, and total cholesterol.

### 2.6. Statistical analyses

The study sample was restricted to participants with non-missing values for exposure, outcome, or any variable used in the adjusted multivariable analyses. We used complex survey methods, including sampling weights, strata, and clusters, to obtain nationally representative estimates for all analyses. To account for the effects of the survey design, Taylor series linearization was used to quantify all the variance values. Missing values related to variance estimation were assumed not to be completely missing at random.

Participant characteristics stratified by race/ethnicity for those with versus without MASLD were examined by testing the difference in means for continuous variables, analysis of variance (ANOVA), and Rao Scott Chi-Square for categorical variables. Age-adjusted mean estimates for clinical characteristics related to MetS were quantified according to sex and race/ethnicity of participants with MASLD. We also evaluated the age-adjusted distributions of MetS severity scores by sex and race/ethnicity for participants with and without MASLD. In addition, we estimated age-adjusted MASLD prevalence by race/ethnicity, sex, and MetS severity Z-score quartile to assess the disease severity distributions.

Multivariable logistic regression models were used to test the association between increased MetS severity and the odds of MASLD. The dose-response relationships between MetS severity and the odds of MASLD were evaluated using the MetS severity score percentiles as a continuous variable, with a three-knot restricted cubic spline (RCS) added to the adjusted logistic regression models. As recommended by Harrell, 2015 [41], the three knots were placed at the 10^th^, 50^th^, and 90^th^ of the weighted MetS severity score percentiles for all participants. Wald chi square tests were used to assess the overall and nonlinear associations of the MetS severity score percentiles with the odds of MASLD. Statistical significance was set at P < 0.05. All analyses were performed using SAS software (version 9.4; SAS Institute, Cary, NC, USA). We followed the Strengthening the Reporting of Observational Studies in Epidemiology (STROBE) reporting guidelines [42].

## 3. Results

### 3.1. Participants baseline characteristics

The baseline characteristics of the adult participants with and without MASLD, according to race/ethnicity, are shown in Table 1. The study sample included 10,605 adult participants from the NHANES III who met the inclusion and exclusion criteria. Independent of race/ethnicity, participants with MASLD were older than their non-MASLD counterparts. Black non-Hispanic and Mexican American adults with MASLD were more likely to be female than those without MASLD. Lower education levels were associated with increased MASLD in white non-Hispanics and Hispanics, but not in Black non-Hispanics. The distributions of alcohol intake and smoking status differed according to the MASLD status, independent of race/ethnicity.

**Table 1:** Sample characteristics by race/ethnicity and Metabolic Dysfunction-Associated Steatotic Liver Disease (MASLD) Status, United States adults, The National Health and Nutrition Examination Survey (NHANES III) 1988–1994 (n = 10,605).

	White, non-Hispanics			Black, non-Hispanics			Mexican Americans		
Characteristics	MASLD	No MASLD	P-value*	MASLD	No MASLD	P-value*	MASLD	No MASLD	P-value*
	(n = 1,029)	(n = 3,075)		(n = 756)	(n = 2,385)		(n = 1,115)	(n = 2,065)	
**Sex,** % (SE)			0.168			0.008			<0.001
Male	46.2 (1.5)	49.1 (0.9)		38.1 (2.2)	44.7 (1.3)		43.3 (1.4)	55.5 (1.2)	
Female	53.8 (1.5)	50.9 (0.9)		61.9 (2.2)	55.3 (1.3)		56.7 (1.4)	44.5 (1.2)	
**Age,** (years)			<0.001			<0.001			<0.001
Median (25^th^, 75^th^ Percentile)	44.2 (33.6, 58.1)	39.4 (29.6, 52.7)		38.1 (28.7, 51.6)	35.7 (27.4, 46.6)		36.7 (28.0, 47.2)	31.6 (24.6, 41.6)	
Mean (SE)	46.3 (0.6)	42.3 (0.5)		41.2 (0.5)	39.1 (0.4)		39.4 (0.6)	35.4 (0.4)	
**Age Group,** % (SE)			<0.001			0.006			<0.001
18–34	26.2 (2.4)	36.9 (1.4)		38.7 (2.1)	44.8 (1.3)		41 (2.1)	57.2 (1.7)	
35–49	32.6 (2.5)	32.8 (1.1)		32.9 (2.1)	32.9 (1.1)		36.6 (1.7)	27.6 (1.5)	
49–64	25.8 (1.6)	19.3 (0.8)		19.1 (1.5)	15.4 (1.1)		15.7 (1.3)	11 (1)	
65+	15.4 (1.1)	11.1 (0.8)		9.3 (1.0)	6.9 (0.7)		6.7 (0.7)	4.2 (0.5)	
**Education Level,** % (SE)			0.001			0.727			0.042
< High School	18.3 (1.4)	16.9 (1.1)		29.2 (2.7)	28.4 (1.5)		59.6 (1.8)	53.5 (1.9)	
High School or GED	40.5 (1.7)	34.0 (1.1)		39.1 (2.7)	39.1 (1.4)		23.7 (1.6)	26.4 (1.2)	
Some College	19.8 (1.7)	23.4 (1.1)		21.9 (1.9)	20.8 (1.1)		11.3 (1.1)	14.6 (1.3)	
College degree or Higher	21.4 (2.0)	25.7 (1.2)		9.8 (1.3)	11.7 (1.2)		5.4 (0.8)	5.4 (0.8)	
**Have Health Insurance,** % (SE)	91.7 (0.9)	89.5 (1.0)	0.088	84.7 (2.1)	83.6 (1.6)	0.428	62.8 (1.8)	61.3 (1.9)	0.443
**Alcohol Intake,** % (SE)			<0.001			<0.001			<0.001
Never	13.6 (1.3)	7.7 (0.8)		21.4 (1.6)	16.1 (1.1)		20.5 (1.7)	15.2 (1.3)	
Former	33.9 (1.7)	30.5 (1.7)		42.1 (1.9)	35.4 (1.5)		34.8 (1.3)	30.8 (1)	
> 0–1 drinks/day	42.1 (2.0)	43.4 (1.6)		29 (2.3)	32 (1.4)		36 (2.9)	36.4 (1.1)	
> 1 drinks/day**	10.4 (1.3)	18.4 (1.1)		7.6 (0.9)	16.5 (0.8)		8.7 (1.3)	17.6 (1)	
**Smoking Status,** % (SE)			<0.001			<0.001			0.017
Never	44.9 (1.6)	41.2 (1.3)		58.6 (2.3)	49.8 (1.1)		61.6 (1.5)	56.2 (1.1)	
Former	31.8 (1.7)	27.1 (0.9)		18.7 (1.5)	14.9 (0.9)		20.4 (1.5)	20.1 (1.2)	
Current	23.3 (1.5)	31.7 (1.2)		22.7 (1.9)	35.3 (1.1)		18.0 (1.3)	23.7 (1.3)	
**Body Mass Index** (Kg/M^2^)			<0.001			<0.001			<0.001
Median (25^th^, 75^th^ Percentile)	27.8 (24.2, 32.0)	24.7 (22.2, 27.8)		28.9 (23.7, 34.6)	26.2 (23.0, 30.1)		28.6 (25.4, 32.2)	25.8 (23.2, 28.7)	
Mean (SE)	28.6 (0.4)	25.5 (0.1)		30.0 (0.4)	27.1 (0.2)		29.3 (0.3)	26.5 (0.1)	
**Body Mass Index Category**^†^ (Kg/M^2^), % (SE)			<0.001			<0.001			<0.001
Underweight	1.9 (0.4)	2.5 (0.4)		2.6 (0.6)	2.2 (0.3)		0.7 (0.3)	1.3 (0.4)	
Healthy Weight	27.1 (2.2)	49.6 (1.1)		27.2 (1.8)	37.3 (1.3)		22.1 (2.3)	40.5 (1.1)	
Overweight	34.1 (1.7)	32.4 (1.0)		26.6 (1.3)	34.2 (0.9)		36.9 (1.7)	38.8 (1.1)	
Obese	36.9 (2.3)	15.6 (1.0)		43.6 (1.9)	26.3 (1.2)		40.3 (2.3)	19.5 (1.1)	
**Waist to Hip Ratio**			<0.001			<0.001			<0.001
Median (25^th^, 75^th^ Percentile)	0.93 (0.85, 0.99)	0.89 (0.83, 0.95)		0.91 (0.85, 0.97)	0.88 (0.82, 0.94)		0.94 (0.88, 0.99)	0.91 (0.85, 0.96)	
Mean (SE)	0.93 (0.004)	0.90 (0.002)		0.89 (0.004)	0.91 (0.002)		0.94 (0.003)	0.92 (0.002)	
**Abdominal Obesity** ^**‡**^, % (SE)	75.5 (1.8)	62.6 (1.3)	<0.001	70.3 (1.9)	57.6 (1.6)	<0.001	83.4 (1.2)	70.1 (1.2)	<0.001
**Physical Activity** (METs/month)			0.002			0.005			0.100
Median (25^th^, 75^th^ Percentile)	62.9 (17.5, 149.5)	78.8 (23.7, 167.5)		39.5 (1.1, 124.5)	52.0 (7.7, 148.8)		22.6 (0, 104.9)	34.7 (4.1, 124.8)	
Mean (SE)	100.6 (4.4)	118.5 (3.9)		88.3 (5.3)	107.9 (4.7)		78.5 (6.1)	89.9 (3.8)	
**Physically Active,** % (SE)	86.2 (1.3)	90.7 (0.7)	<0.001	75.7 (2.5)	80.5 (1.2)	0.035	72.1 (2.1)	76.9 (1.5)	0.008
**Healthy Eating Index**			0.470			0.390			0.102
Median (25^th^, 75^th^ Percentile)	64.5 (54.1, 74.0)	63.7 (54.5, 73.2)		60.2 (50.6, 68.7)	58.7 (50.2, 67.8)		65.3 (56.0, 73.9)	63.8 (55.7, 72.2)	
Mean (SE)	64.1 (0.5)	63.8 (0.4)		59.5 (0.6)	58.9 (0.2)		64.8 (0.6)	63.8 (0.6)	
**Healthy Eating Index** ^§^, % (SE)			0.369			0.517			0.449
Poor	17.2 (1.3)	16.6 (0.9)		25.4 (2.1)	27.4 (0.7)		14.2 (1.6)	15.3 (1.2)	
Fair	69.8 (1.8)	71.9 (0.7)		70 (2.0)	67.6 (0.8)		73.7 (1.6)	74.2 (1.0)	
Good	13.0 (1.4)	11.5 (0.7)		4.6 (0.7)	5.1 (0.5)		12.1 (1.3)	10.5 (1.2)	
**Total Cholesterol (mg/dL)**			0.006			0.002			0.015
Median (25^th^, 75^th^ Percentile)	204.7 (176.9, 235.6)	199.0 (173.3, 227.7)		202.6 (172.8, 231.5)	192.5 (168.2, 220.8)		198.2 (170.8, 228.2)	190.9 (166.2, 219.7)	
Mean (SE)	208.4 (1.7)	202.8 (1.0)		205.6 (1.8)	197.2 (1.0)		201.9 (2.4)	195.2 (1.7)	
**HOMA-IR**			<0.001			<0.001			<0.001
Median (25^th^, 75^th^ Percentile)	2.39 (1.58, 3.90)	1.62 (1.19, 2.32)		3.09 (1.80, 5.38)	2.09 (1.41, 3.18)		3.06 (1.97, 5.0)	1.92 (1.32, 3.03)	
Mean (SE)	3.9 (0.3)	2.2 (0.08)		5.2 (0.3)	3.4 (0.2)		4.9 (0.4)	2.8 (0.1)	
**AST/ALT Ratio**			<0.001			0.004			<0.001
Median (25^th^, 75^th^ Percentile)	1.20 (0.92, 1.54)	1.35 (1.06, 1.69)		1.38 (1.09, 1.78)	1.49 (1.18, 1.89)		1.05 (0.79, 1.35)	1.23 (0.95, 1.54)	
Mean (SE)	1.3 (0.04)	1.5 (0.08)		1.5 (0.04)	1.6 (0.04)		1.1 (0.02)	1.3 (0.02)	

* Rao-Scott Chi Square P-values for difference in proportions and T-tests P-values for difference in means between adults with versus without Metabolic Dysfunction-Associated Steatotic Liver Disease of the same race/ethnicity

** In MASLD up to 2 drink per days for females and 3 drinks per day for males

† Underweight (< 18.50), Healthy Weight (≥ 18.50–25.00 <), Overweight (≥ 25.00–30.00 <) and Obese (≥ 30)

‡ Waist to Hip Ratio ≥ 0.90 for males or ≥ 0.85 for females

§ Poor < 51%, Fair < 80%, Good ≥ 80%

MET = Metabolic equivalent; AST = Aspartate Aminotransferase; ALT = alanine aminotransferase; % = Weighted Proportion; SE = Standard Error

The prevalence estimates for overweight or morbid obesity were 71.0%, 70.0%, and 77.2, respectively, in white non-Hispanics, black non-Hispanics, and Mexican Americans with MASLD, compared to 48.0%, 60.5%, and 58.3%, respectively, in those without MASLD of the same race/ethnicity. Similarly, participants with MASLD had a 3.1 kg/m^2^ higher average BMI than those without MASLD. The prevalence of abdominal obesity was also significantly associated with MASLD status in all racial/ethnic groups. The mean values of total cholesterol and HOMA-IR were higher in the MASLD group than in the no MASLD. In contrast, the proportion of physically active participants was lower in the MASLD group than in the no MASLD, independent of race/ethnicity (Table 1).

### 3.2. MASLD prevalence

The overall prevalence of MASLD was 26.7% (95% CI: 24.3% to 29.1%). Stratified by race/ethnicity, the prevalence of MASLD was 26.7%, 23.3%, and 33.7% in white non-Hispanics, black non-Hispanics, and Mexican Americans, respectively. The prevalence of MASLD was marginally different among males compared to females (25.6% vs. 28.3%; P = 0.06). However, among male participants, the age-adjusted prevalence of MASLD was significantly higher in Mexican Americans than in White non-Hispanics (31.5% vs. 25.9%; P = 0.04). Similarly, Mexican American females had a significantly higher age-adjusted MASLD prevalence than white non-Hispanic females (41.1% vs. 27.6%; P<0.001).

### 3.3. Metabolic syndrome severity in MASLD

The prevalence of traditionally defined MetS was higher in patients with MASLD than in those without (44.0% vs. 20.4%; P-value <0.001). Black non-Hispanic males and females with MASLD had significantly lower age-adjusted MetS prevalence than white non-Hispanic males and females with MASLD (Table 2). Among females with MASLD, Mexican Americans had a significantly higher prevalence of MetS than White non-Hispanics (35.6% vs. 23.7%; P-value <0.001). An estimated 82.1% of adults with MASLD had at least one feature of traditionally defined MetS, whereas 9.2% met the criteria for all five metabolic components. Stratified by race/ethnicity, the prevalence of at least one metabolic abnormality in participants with MASLD was 82.3%, 77.2%, and 86.9% in White non-Hispanics, Black non-Hispanics, and Mexican Americans, respectively. The age-adjusted average number of metabolic abnormalities was highest in Mexican American females with MASLD (2.1) and lowest in Black non-Hispanic males (1.4).

**Table 2:** Age adjusted estimates for clinical characteristics related to metabolic syndrome by sex and race/ethnicity, adults with Metabolic Dysfunction-Associated Steatotic Liver Disease (MASLD), The National Health and Nutrition Examination Survey (NHANES III) 1988–1994 (n = 3,080).

Clinical Characteristics	Males			Females		
	White, non-Hispanics	Black, non-Hispanics	Mexican Americans	White, non-Hispanics	Black, non-Hispanics	Mexican Americans
	(n = 1,961)	(n = 1,351)	(n = 1,553)	(n = 2,323)	(n = 1,790)	(n = 1,627)
**Number of Metabolic Abnormalities**^**†**^, Mean (SE)	1.8 (0.04)	1.5 (0.04) *	1.9 (0.05) *	1.5 (0.06)	1.8 (0.03) *	2.1 (0.03) *
**Metabolic Syndrome**^**‡**^**, %** (SE)	27.9 (0.01)	21.4 (0.01) *	31.1 (0.01)	23.7 (0.01)	27.7 (0.01) *	35.6 (0.01) *
**Central Obesity,** % (SE)	31.1 (0.01)	26.6 (0.01) *	31.1 (0.02)	40.8 (0.01)	59.5 (0.01) *	60.4 (0.01) *
Waist Circumference (inches), Mean (SE)	96.6 (0.3)	93.3 (0.3) *	96.1 (0.4)	87.7 (0.5)	93.9 (0.5) *	93.1 (0.4) *
**Hypertriglyceridemia,** % (SE)	40.8 (0.02)	27.0 (0.01) *	47.4 (0.02) *	26.7 (0.01)	19.6 (0.01) *	39.6 (0.01) *
Triglyceridemia (mg/dL), Mean (SE)	161.0 (3.9)	128.5 (2.6) *	182.1 (4.4) *	130.8 (3.4)	111.1 (2.0) *	153.8 (3.2) *
**Hypertension,** % (SE)	38.0 (0.01)	46.1(0.01) *	36.8 (0.01)	30.3 (0.01)	43.3 (0.01) *	35.5 (0.01) *
SBP (mmHg), Mean (SE)	106.1 (0.8)	108.6 (0.8) *	106.0 (0.8)	87.3 (0.6)	93.1 (0.6) *	89.8 (0.6) *
**Dyslipidemia,** % (SE)	38.2 (0.02)	24.2 (0.01) *	37.4 (0.02)	38.5 (0.02)	36.3 (0.01)	47.8 (0.02) *
HDL (mg/dL), Mean (SE)	44.8 (0.5)	52.1 (0.5) *	45.3 (0.5)	55.6 (0.6)	57.1 (0.5) *	52.1 (0.5) *
**Hyperglycemia,** % (SE)	30.4 (0.01)	30.4 (0.01)	38.2 (0.02) *	17.8 (0.01)	25.9 (0.01) *	30.5 (0.01) *
Plasma Glucose (mg/dL), Mean (SE)	100.1 (0.6)	101.9 (0.8)	105.8 (0.9) *	95.1 (0.7)	102.0 (1.5) *	102.2 (0.7) *

HDL, High-Density Lipoprotein Cholesterol; SBP, Systolic Blood Pressure

* P-value <0.05 for adults of the same sex in reference to white non-Hispanics

† (1) hyperglycemia (i.e., fasting blood glucose over 100 mg/dl, or pharmacological treatment), (2) dyslipidemia (i.e., fasting HDL cholesterol level less than 40 mg/dl, men, or 50 mg/dl, women, or pharmacological treatment) (3) hypertriglyceridemia (i.e., fasting triglyceride (TG) level over 150 mg/dl, or pharmacological treatment), (4) central obesity (i.e., waist circumference over 40 inches, men, or 35 inches, women), or (5) hypertension (i.e., systolic blood pressure (SBP) over 130 mmHg, or pharmacological treatment)

^**‡**^ Three or more metabolic abnormalities

We observed racial/ethnic disparities in the age-adjusted prevalence of all five metabolic abnormalities in male and female patients with MASLD (Table 2). In males with MASLD, the age-adjusted prevalence of central obesity, hypertriglyceridemia, and dyslipidemia was lower in black non-Hispanics than in white non-Hispanics. In contrast, the age-adjusted prevalence of hypertension was higher in male black non-Hispanic patients with MASLD than in their white non-Hispanic counterparts. Mexican American males with MASLD had a significantly higher age-adjusted prevalence of hypertriglyceridemia and hyperglycemia than white non-Hispanic males with MASLD.

Both black non-Hispanic and Mexican American females with MASLD had a higher age-adjusted prevalence of central obesity, hypertension, and hyperglycemia than white non-Hispanic females with MASLD. Furthermore, Mexican American females with MASLD had a higher age-adjusted prevalence of hypertriglyceridemia and dyslipidemia than white non-Hispanic females with MASLD. Similar to Black non-Hispanic males, black non-Hispanic females had a lower age-adjusted hypertriglyceridemia prevalence than white non-Hispanic females with MASLD.

MetS severity was normally distributed in the MASLD and no-MASLD groups. However, the severity of MetS was significantly higher in the patients with MASLD. The overall mean and median MetS severity scores and corresponding percentiles were 0.03 (49^th^) and -0.07 (47^th^), respectively. Both the mean and median MetS severity scores were significantly higher in MASLD relative to those without [mean MetS severity Z-score, 0.48 (61^st^) vs. -0.14 (46^th^); median MetS severity Z-score, 0.48 (69^th^) vs. -0.23 (41^st^)].

**Fig 1:**
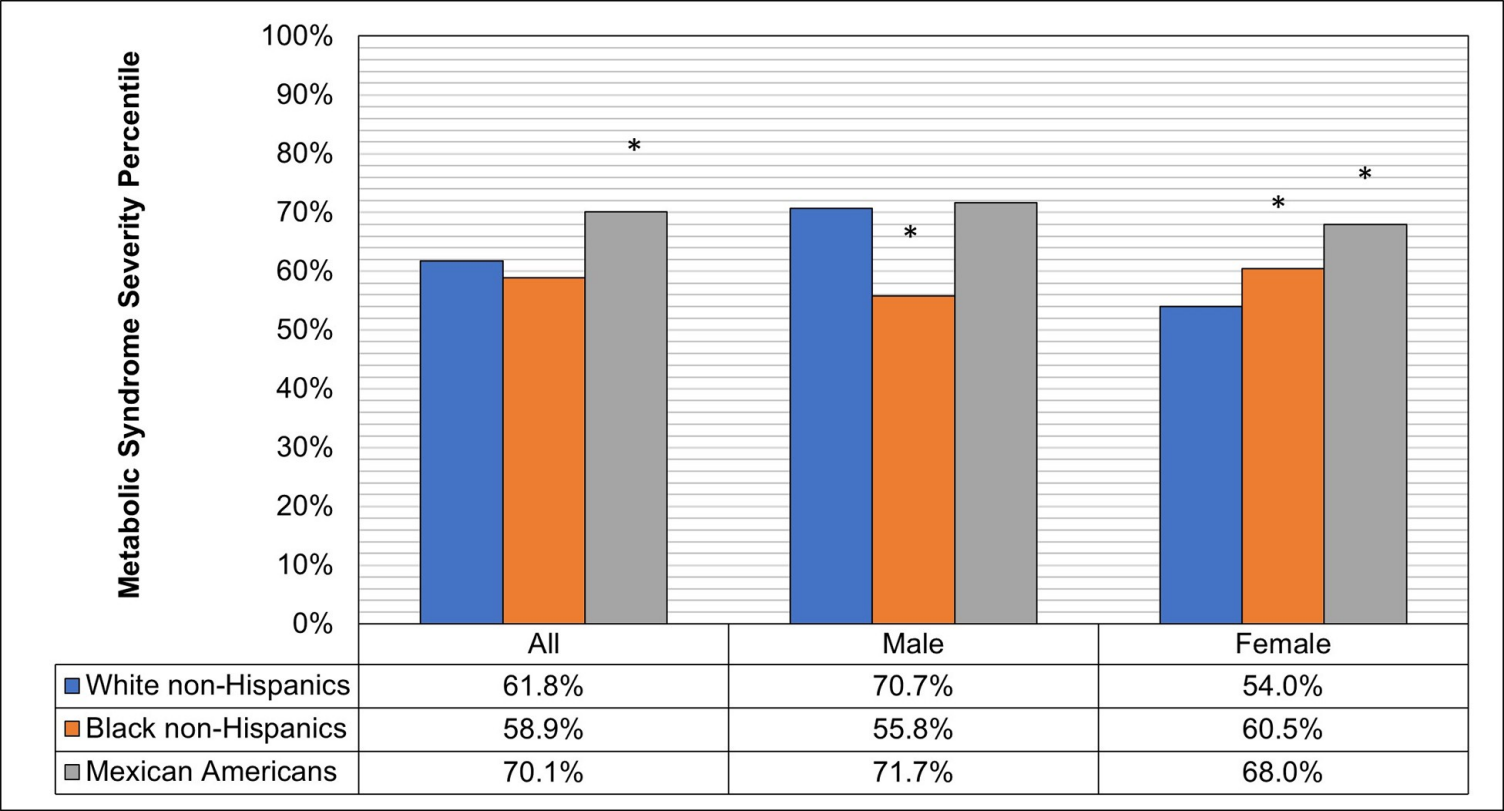
Age adjusted mean metabolic syndrome severity percentile by race/ethnicity and sex in adults with Metabolic Dysfunction-Associated Steatotic Liver Disease, The National Health and Nutrition Examination Survey (NHANES III), United States, 1988–1994 (n = 3,088). * P-value <0.05 for the difference in metabolic syndrome percentile in reference to white non-Hispanics of the same sex.

When the sample was stratified by race/ethnicity, and sex, patients with MASLD had significantly higher MetS severity in all comparison groups. Among adults without MASLD, Mexican Americans had the highest severity (53^rd^) compared with both white non-Hispanics (45^th^) and black non-Hispanics (43^rd^). Among participants with MASLD, Mexican American and Black non-Hispanic females had significantly higher MetS severity than White non-Hispanic females (Fig 1). Black non-Hispanic males with MASLD had significantly lower MetS severity (56^th^ percentile) than white non-Hispanic males with MASLD (70^th^ percentile).

### 3.4. MASLD and metabolic syndrome severity

**Fig 2:**
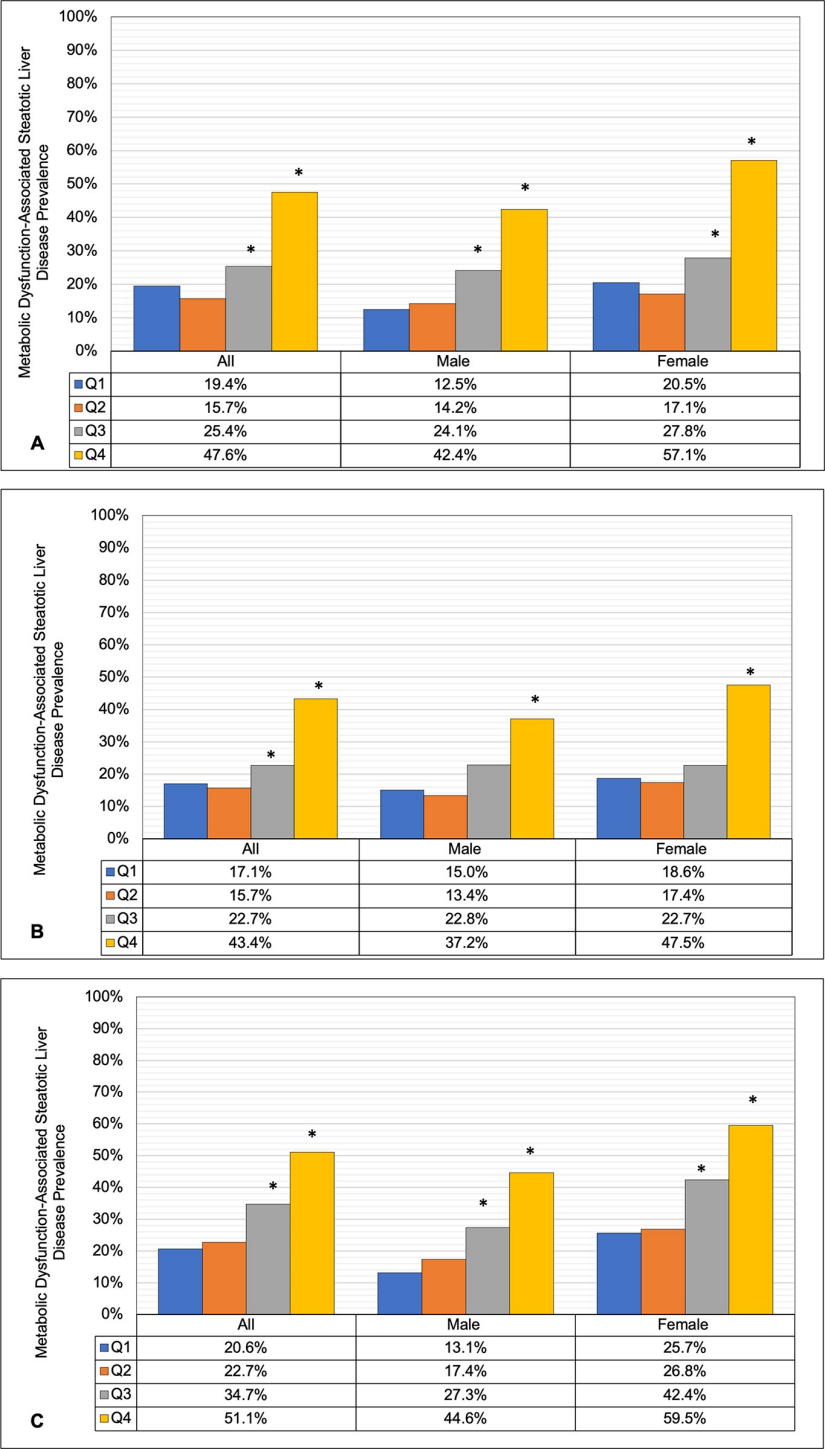
Age adjusted Metabolic Dysfunction-Associated Steatotic Liver Disease Prevalence by Sex and Metabolic Syndrome Severity Z-Score Quartile (Q) for A) White, non-Hispanics B) Black, non-Hispanics and C) Mexican Americans, Adult Participants in The National Health and Nutrition Examination Survey (NHANES III), United States, 1988–1994 (n = 10,605). * P-value <0.05 for the difference in age adjusted Metabolic Dysfunction-Associated Steatotic Liver Disease prevalence relative to the first severity quartile.

The age-adjusted prevalence of MASLD increased significantly with higher MetS severity group (P-trend <0.001). When the MetS severity score was divided into quartiles, the age-adjusted MASLD prevalence was not significantly different between the first and second MetS severity quartiles (Fig 2A to 2C). In contrast, those in the third and fourth MetS severity quartiles had a significantly higher age-adjusted MASLD prevalence than those in the first severity quartile, independent of race/ethnicity and sex. Similarly, the prevalence of MASLD in the second and third severity quartiles was higher than that in the first quartile among White non-Hispanic and Mexican American males and females (Fig 2A and 2C). In both Black non-Hispanic males and females, only the third severity quartile was significantly higher than the first quartile (Fig 2B).

### 3.5. Metabolic syndrome severity and the odds of MASLD occurrence

The MetS severity score significantly predicted the occurrence of MASLD in all crude and adjusted regression models. A quartile increase in the MetS severity score was associated with a 36% increase in the adjusted odds for MASLD aOR 1.36 (95% CI:1.17 to 1.57). Similarly, a ten-percentile increase in MetS severity was associated with a 1.15 (95% CI:1.09 to 1.20) higher adjusted odds of MASLD. In the adjusted models, with the severity score included as a categorical variable, adults with high MetS severity had aOR 2.27 (95% CI; 1.70 to 3.03) times the odds of MASLD relative to those with mild MetS severity score (Table 3). Very high MetS severity was associated with 3.12 (95% CI; 2.20 to 4.42) higher adjusted odds of MASLD than mild MetS severity.

**Table 3:** The association between metabolic syndrome severity and odds of Metabolic Dysfunction-Associated Steatotic Liver Disease Occurrence in United States adults, the National Health and Nutrition Examination Survey (NHANES III) 1988–1994 (n = 10,605).

Metabolic Syndrome Severity	Unadjusted	Model 1	Model 2
	OR (95% CI)	OR (95% CI)	OR (95% CI)
Low	Reference	Reference	Reference
Moderate	1.69 (1.36 ⎯ 2.11)	1.80 (1.45 ⎯ 2.24)	1.26 (0.97 ⎯ 1.64)
High	3.67 (2.96 ⎯ 4.54)	3.99 (3.20 ⎯ 4.98)	2.27 (1.70 ⎯ 3.03)
Very High	6.08 (4.79 ⎯ 7.72)	6.60 (5.21 ⎯ 8.36)	3.12 (2.20 ⎯ 4.42)

Metabolic Syndrome Severity Group, the Metabolic Syndrome Z-score was transformed into four percentiles-based categories [low (0 – 50th), Moderate (>50th– 75th), High (>75th– 90th), and very-high (>90th)]

Model 1 = adjusted for age, sex, and race/ethnicity

Model 2 = Adjusted for age, sex, race/ethnicity, education level, access to health insurance, alcohol intake, smoking status, body mass index, abdominal obesity, physical activity, healthy eating index percentile, HOMA-IR, total cholesterol, and Aspartate Aminotransferase (AST)/alanine aminotransferase (ALT) Ratio

OR; odds ratio, CI; Confidence interval

In the RCS analysis, a significant nonlinear dose-response trend was observed in the relationship between increased odds of MASLD occurrence and higher MetS severity scores in all adjusted models. Generally, the adjusted odds of MASLD increased with higher MetS severity scores relative to the median severity value (50^th^ percentile). Specifically, compared to those with median severity scores, the aOR of MASLD was 1.17 (95% CI; 1.11 to 1.23), 2.05 (95% CI; 1.72 to 2.43), 2.85 (95% CI; 2.23 to 3.65), and 3.38 (95% CI; 2.55 to 4.49), respectively, for adults in the 60^th^, 80^th^, 90^th^, and 95^th^ severity percentiles (Fig 4). In contrast, the adjusted odds of MASLD were significantly lower for those with MetS severity scores below the median value, up to the 43^rd^ percentile.

**Fig 3:**
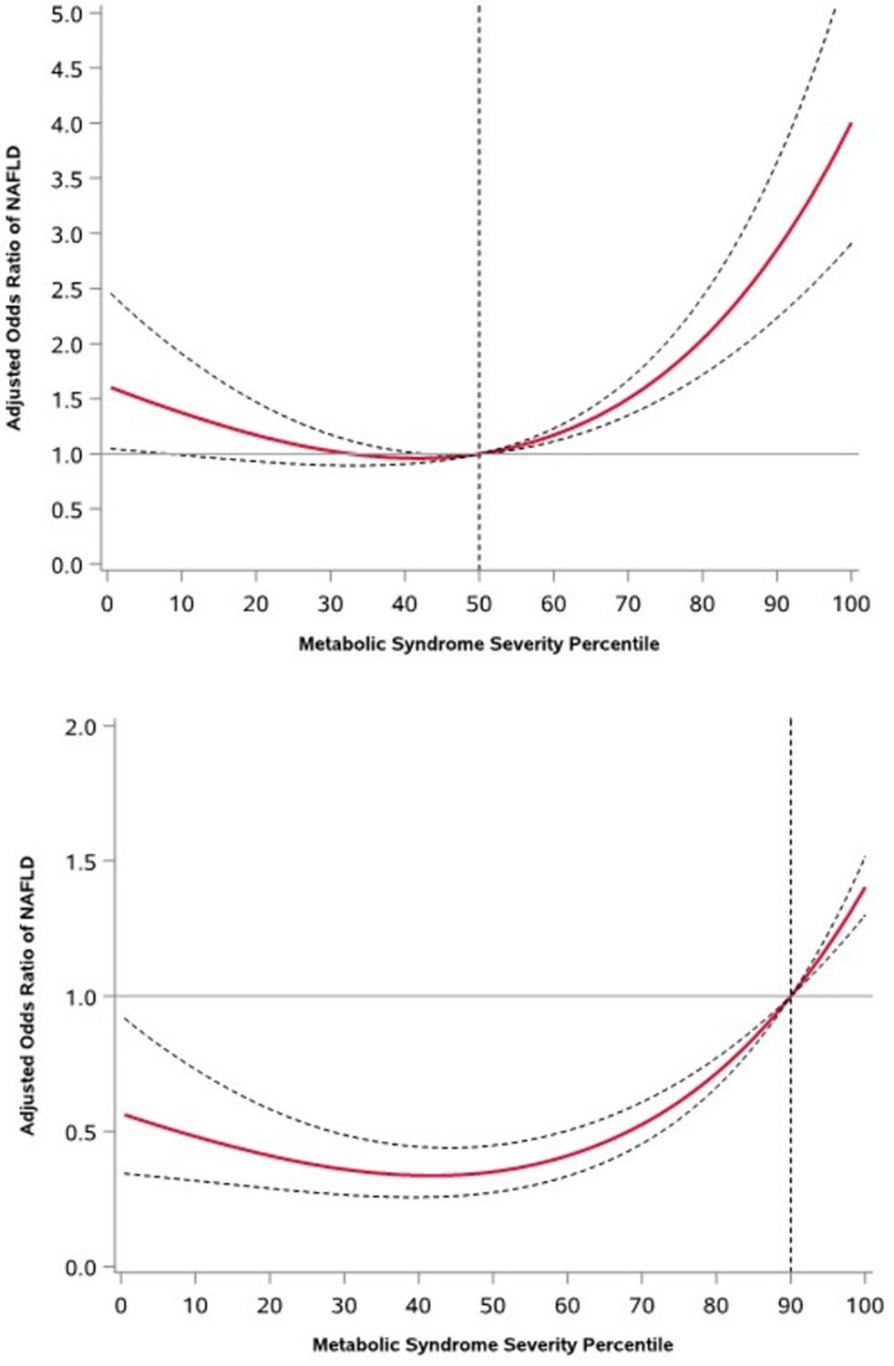
Adjusted Odds Ratios of Metabolic Dysfunction-Associated Steatotic Liver Disease (MASLD) Prevalence for Different Metabolic Syndrome Severity Score Percentiles Relative to A) 50^th^ and B) 90^th^ Severity Percentile as the Reference Levels, the National Health and Nutrition Examination Survey (NAHNES III), United States, 1988–1994 (n = 10,605).

The dose-response relationship between MetS severity and the odds of MASLD was estimated with reference to the 90^th^ severity score percentile (Fig 3B). The adjusted odds of MASLD presence were significantly higher for adults with severity above the 90^th^ percentile relative to those in the 90^th^ percentile. For example, adults in the 95^th^ percentile had aOR 1.18 (95% CI; 1.14 to 1.23) higher odds of MASLD than those in the 90^th^ percentile. In contrast, adults with values below the 90^th^ percentile had significantly lower odds of having MASLD. Relative to those in the 90^th^ percentile, the aOR of MASLD were 0.72 (95% CI; 0.66 to 0.77), 0.35 (95% CI; 0.27 to 0.45), and 0.48 (95% CI; 0.32 to 0.73)] for adults in the 80^th^, 50^th^, and 10^th^ percentiles, respectively.

### 3.6. Prevention paradox

**Fig 4:**
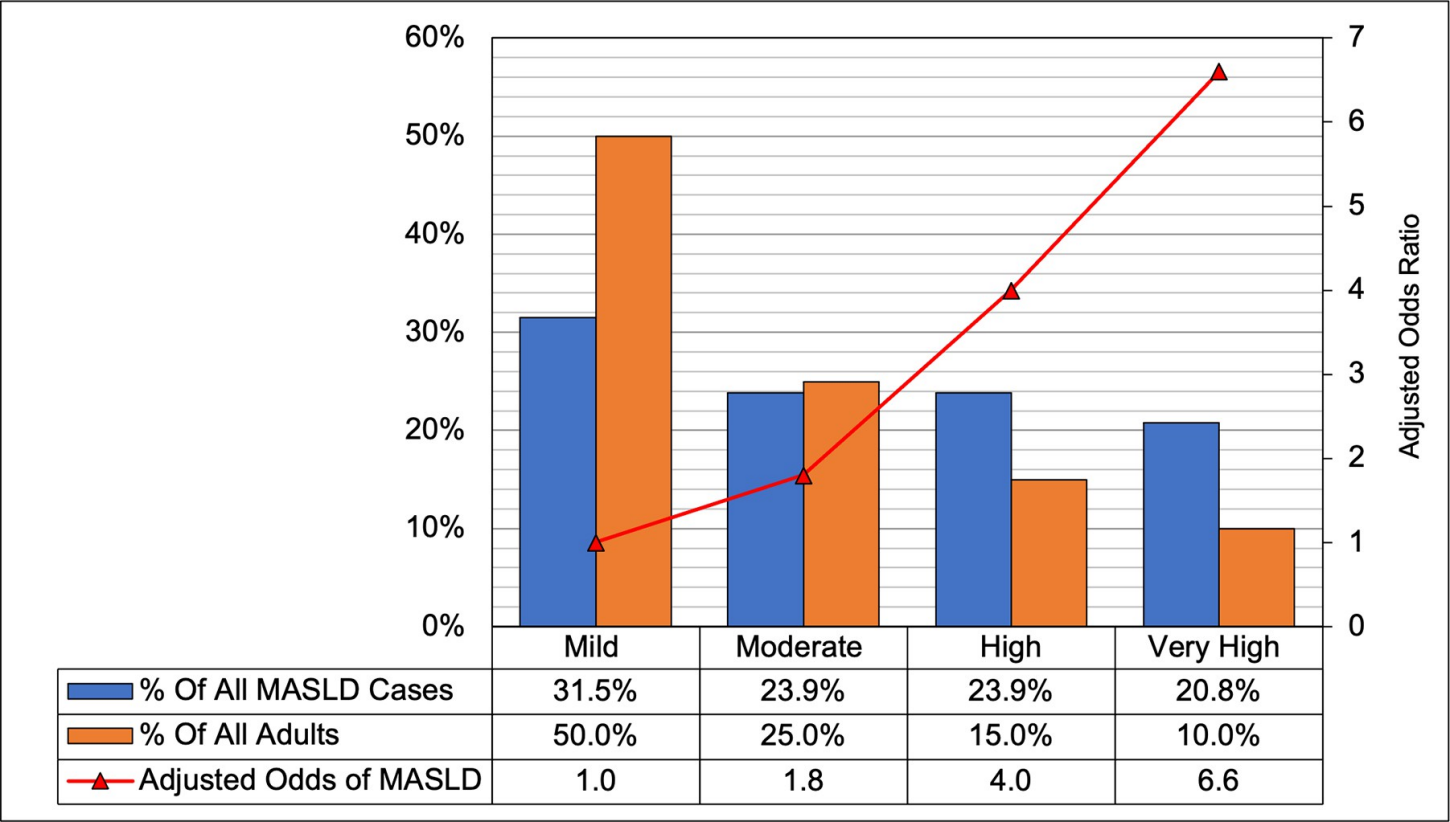
The distribution of metabolic syndrome severity, Metabolic Dysfunction-Associated Steatotic Liver Disease (MASLD) prevalence and the adjusted odds of MASLD by metabolic syndrome severity, United States adults, the Third National Health and Nutrition Examination Survey (NHANES III), 1988–1994 (n = 10,605). * To construct the Metabolic Syndrome Severity Group, the Metabolic Syndrome Z-score was transformed into four percentiles-based categories [low (0 – 50th), Moderate (>50th– 75th), High (>75th– 90th), and very-high (>90th)]. ** MASLD odds ratios in reference to mild severity adjusted for age, sex, and race/ethnicity.

The proportion of participants with mild, moderate, high, and very high MetS severity scores was 50%, 25%, 15%, and 10%, respectively. In patients with mild (0^th^-50^th^), moderate (>50^th^-75^th^), high (>75^th^-90^th^), and very high (>90^th^) MetS severities, the age-adjusted MASLD prevalence was 17.4%, 25.7%, 42.5, and 54.9%, respectively. Approximately 31% of all patients with MASLD had low MetS severity, whereas an estimated 21% had very high severity scores (*i*.*e*., 90^th^ or above) (Fig 4). A dose-response relationship was observed, whereby an increase in the MetS severity group, in reference to mild severity, was associated with higher odds of MASLD presence (Fig 4). The highest odds of MASLD were observed for those with very high versus low MetS severity (aOR, 6.60; 95% CI; 5.21 8.36).

## 4. Discussion

Studies on the link between MetS and MASLD have employed a harmonized disease definition, which encompasses exceeding predefined cutoff values for three out of five metabolic features. This definition accounts for disease occurrence, but equally treats the effects of any combination of metabolic abnormalities on MASLD risk. The traditional definition of MetS also overlooks racial/ethnic and sex disparities in MetS severity and their influence on the risk of MASLD. To bridge this knowledge gap, we used the MetS severity score, a validated sex-race/ethnicity-specific measure, to assess the association between MetS severity and MASLD.

Similar to previous studies, our MASLD cohort was older, with a higher prevalence of morbid obesity and lower physical activity levels than the general US adult population [43, 44]. In MASLD, racial/ethnic disparities were detected in baseline characteristics related to MetS. The prevalence of both MASLD and traditionally defined MetS differed by race/ethnicity, with Mexican American women having the highest disease burden. The severity of MetS was significantly higher in patients with MASLD than in those without. Among those with MASLD, Mexican American male, and female adults had the highest MetS severity compared to white non-Hispanic males and females. The MetS severity score significantly predicted the occurrence of MASLD in all the crude and adjusted models. We also observed nonlinear dose-response relationships between increased adjusted MASLD odds and higher MetS severity scores.

Several studies have examined racial and ethnic MASLD disparities in the context of the prevalence and severity of the individual components of MetS [18, 32, 45–48]. Walker *et al*. studied waist circumference, blood pressure, triglycerides, HDL cholesterol, and fasting glucose measurements in both adolescents and adults, and found that the odds of MetS varied considerably among white non-Hispanics, black non-Hispanics, and Mexican American groups [48]. The prevalence of high blood pressure, elevated fasting glucose, and insulin resistance was significantly higher in male African Americans than in whites. In females, triglycerides were associated with waist circumference in whites but not in African Americans, while African-American women displayed higher prevalence rates of elevated blood pressure, low HDL, and elevated fasting glucose. Genetic and metabolic factors have been suggested to underlie these disparities [25, 28, 49], as well as the incidence rates of insulin resistance and serum triglyceride concentrations [50, 51]. However, conclusive evidence supporting any model explaining race/ethnic disparities in MASLD is yet to be uncovered. Our study expands on these findings by accounting for the combined effects of all five metabolic features and their severities to explain racial/ethnic disparities in MASLD.

Our findings showed that increased MetS severity was associated with higher nonlinear odds of developing MASLD. This nonlinear relationship between MetS severity and the odds of MASLD helps explain the hepatic progression dynamics in MASLD. The onset of obesity is associated with the excessive accumulation of triglycerides throughout the body. In hepatocytes, increased uptake of triglycerides results in cell-specific lipotoxicity, which elevates the risk of comorbidities such as MASLD [52]. Individuals with high visceral adiposity may suffer from increased plasma-free fatty acids due to impaired insulin function related to peripheral Insulin resistance [53]. Due to the central role that obesity-induced insulin resistance plays in promoting hepatic steatosis, MASLD is regarded as the hepatic manifestation of MetS [4, 7, 11–13, 54, 55]. The prevalence of obesity among MASLD and MASH patients is 51% and 82%, respectively, and the prevalence of MASLD in patients with MetS and diabetes is particularly high [28, 56]. In the absence of MetS or any of its components, the prevalence of MASLD was 6.1% [39].

Our current study aligns with and extends the findings from our previous work on the relationship between MetS severity and mortality risk in patients with NAFLD [31]. In this work, MetS severity was markedly higher in NAFLD patients, indicating a significant predictor for all-cause and cause-specific mortalities. This retrospective cohort study, with a median follow-up of 19.2 years, showed that increases in MetS severity were associated with dose-response increases in biomarkers for cardiovascular disease, insulin resistance, and lipid abnormalities. Additionally, significant nonlinear dose-response relationships were found between increased adjusted mortality risk and higher MetS severity scores. These findings underscore the importance of MetS severity as a driving force for increased mortality risk, thereby reinforcing the need for its incorporation into risk stratification and management strategies for patients with MASLD. Echoing findings from this work, our results further affirm the utility of the MetS severity score as an instrumental clinical tool for the identification and longitudinal monitoring of at-risk patients, which is paramount given the currently limited treatment options for MASLD.

Approximately 55% of all patients with MASLD in our weighted sample had low-to-moderate MetS severity, whereas 21% had very high severity scores (*i*.*e*., 90^th^ or above). Therefore, effective prevention measures must combine distinct strategies for high-risk individuals and the general population. Patients with very high MetS severity are likely to seek medical care because of the increased disease severity. Furthermore, high-risk adults are prone to develop advanced symptomatic hepatic conditions, such as MASH, fibrosis, cirrhosis, and HCC. Hence, preventive measures for this group are expected to be secondary and tertiary. Early detection and diagnosis of those at the highest risk will ensure increased survival by mitigating hepatic progression and lowering the elevated risks of CVD and cancer. While pharmaceutical intervention for MASLD must provide substantial clinical benefits at a modest annual price to be cost-effective [57], bariatric surgery has been shown to reduce the risk of cancer and cardiovascular disease in MASLD patients with morbid obesity [58, 59].

A population-wide preventive strategy should employ primary prevention measures, such as promoting a healthy lifestyle and dietary changes, to reduce MetS severity. While the natural history of MASLD might extend beyond the MetS spectrum, those with low-to-moderate severity are less likely to seek MASLD-related medical interventions. Thus, regardless of the causal mechanisms linking low to moderate severity with MASLD, low severity could be viewed as a proxy for a lower likelihood of seeking medical care, resulting in an increased risk of disease progression. From a policy perspective, primary interventions should attempt to shift the mean MetS severity score for patients with MASLD downwards. Because MetS severity predicts a wide array of cardiovascular outcomes, an overall decrease in MetS severity will provide significant public health benefits beyond the MASLD spectrum.

Our study has several limitations. The MASLD assessment was performed using ultrasonography, which could have resulted in misclassification. However, ultrasonography images were assessed by three trained ultrasound readers using standardized reading protocols. Furthermore, due to the lack of data, we could not evaluate the role of MASH, MASH-cirrhosis, and MetS severity. However, MASH and MASH-cirrhosis were associated with higher MetS severity, which is expected to reaffirm our findings on the association between MetS severity and MASLD occurrence. Ascertainment of exposure and baseline characteristics were conducted cross-sectionally, which resulted in nondifferential measurements in the exposed and unexposed groups. Alcohol intake was assessed based on self-reporting, which might have resulted in underestimation.

In conclusion, we have highlighted the pivotal role of MetS severity in driving the risk and racial/ethnic disparities observed in MASLD among adults in the US. The nonlinear dose-response relationship between increased MetS severity and higher odds of MASLD emphasizes the importance of considering disease severity rather than mere presence in understanding MASLD development and progression. Given the distribution of MetS severity in the MASLD spectrum, customized prevention strategies should be considered to address this hepatic epidemic. While the current treatment options for patients with MASLD are limited and indirect, the MetS severity score is a clinically accessible tool that can be used in primary and secondary prevention efforts to reduce the disease burden.
